# Correction: The views, perspectives, and experiences of academic researchers with data sharing and reuse: A meta-synthesis

**DOI:** 10.1371/journal.pone.0234275

**Published:** 2020-06-02

**Authors:** The PLOS ONE Staff

## Notice of republication

This article was republished on May 8, 2020, to correct an error in the heading levels in the Findings subsection of the Results. Please download the article again to view the correct version. The originally published, uncorrected article and the republished, corrected article are provided here for reference.

## Supporting information

S1 File Originally published, uncorrected article. (PDF)

S2 File Republished corrected article. (PDF)
