# Supplementary material for: The views, perspectives, and experiences of academic researchers with data sharing and reuse: A meta-synthesis
Source: PLoS One. 2020 Feb 27;15(2):e0229182. doi: 10.1371/journal.pone.0229182 (PMC7046208; doi:10.1371/journal.pone.0229182)
Supplement: S3 Appendix — (DOCX) [file pone.0229182.s003.docx]

## S3 Appendix. Literature databases searched.

| **Literature database** | **Discipline or area of study** | **Number of included studies identified,  *n* (%); *N* = 45** ^a,b^ | **Classification of discipline** ^c^ |
| --- | --- | --- | --- |
|  |  |  |  |
| MEDLINE (OVID) | Bioethics; Biophysics; Dentistry; Forensic Science; Genetics; Gerontology and Geriatrics; Health Administration; Kinesiology; Medicine; Neuroscience; Nursing; Nutrition; Occupational Therapy; Pharmacology; Pharmacy; Physical Education; Physical Therapy; Physiology; Psychiatry; Public Health; Rehabilitation Sciences; Speech Language Pathology; Toxicology; Veterinary Sciences | 8 (17%) | Applied Science |
| Compendex (Engineering Village) | Aerospace; Biotechnology; Energy; Engineering; Food Science; Materials Science; Transportation | 8 (17%) | Applied Science |
| INSPEC (Engineering Village) | Aerospace; Astronomy; Biophysics; Computer Science; Energy; Engineering; Physics | 4 (8%) | Applied Science / Basic Science |
| Library and Information Science Abstracts (LISA) (ProQuest) | Library and Information Science | 6 (13%) | Applied Science |
| BIOSIS Previews (Web of Science) | Agriculture; Aquatic Sciences; Biochemistry; Bioethics; Biology; Biophysics; Botany; Food Science; Genetics; Medicine; Microbiology; Neuroscience; Pharmacology; Physiology; Veterinary Sciences | 3 (6%) | Applied Science / Basic Science |
| ABI/INFORM Complete (ProQuest) | Business; Finance | 3 (6%) | Social Science |
| ERIC (ProQuest) | Education; Kinesiology; Physical Education; Speech Language Pathology | 1 (2%) | Social Science / Applied Science |
| SciFinder Scholar | Biochemistry; Chemistry; Engineering; Food Science; Forensic Science; Materials Science; Pharmacology | 2 (4%) | Applied Science / Basic Science |
| PsycINFO (ProQuest) | Criminology; Forensic Science; Gerontology and Geriatrics; Kinesiology; Medicine; Neuroscience; Physical Education; Psychiatry; Psychology; Speech Language Pathology; Substance Abuse | 2 (4%) | Social Science |
| General Science Abstracts (H.W. Wilson) | Astronomy; Science | - | Basic Science |
| *Replacement for update:* Web of Science Core Collection | Astronomy; Science | - | Basic Science |
| CAB Abstracts (OVID) | Agriculture; Environment; Food Science; Forestry; Kinesiology; Nutrition; Recreation; Sports | 4 (8%) | Applied Science |
| Social Sciences Abstracts (H.W. Wilson) | Anthropology; Geography; Social Sciences; Social Work; Sociology; Sports; Urban Studies | - | Social Science |
| Sociological Abstracts (ProQuest) | Criminology; Equity Studies; Middle Eastern Studies; Sociology; Urban Studies; Women and Gender Studies | - | Social Science |
| PAIS International (ProQuest) | Economics; International Development; International Relations; Islamic Studies; Middle Eastern Studies; Political Science; Public Health; Slavic and East European Studies | - | Social Science |
| ASFA: Aquatic Sciences and Fisheries Abstracts (ProQuest) | Aquatic Sciences; Zoology | 1 (2%) | Basic Science |
| Environmental Sciences and Pollution Management (ProQuest) | Aquatic Sciences; Ecology; Environment | - | Applied Science |
| *Replacement for update:* GREENFile | Environment; Pollution; Renewable Energy | - | Applied Science |
| Philosopher’s Index (ProQuest) | Bioethics; Islamic Studies; Philosophy | - | Humanities |
| Accounting & Tax (ProQuest) | Accounting | - | Social Science |
| *Replacement for update:*  Business Source Premier (EBSCO) | Accounting | - | Social Science |
| Applied Social Sciences Index and Abstracts (ASSIA) (ProQuest) | Criminology; Forensic Science; Gerontology and Geriatrics; Health Administration; Nursing; Occupational Therapy; Psychology; Public Health; Rehabilitation Sciences; Social Sciences; Social Work; Sociology; Substance Abuse; Transportation; Urban Studies; Women and Gender Studies | - | Applied Science / Social Science |
| ARTbibliographies Modern (ABM) (ProQuest) | Architecture | - | Arts |
| Avery Index to Architectural Periodicals (ProQuest) | Architecture; Landscape Architecture | - | Arts |
| British Humanities Index (ProQuest) | Celtic Studies; Humanities; History | - | Humanities |
| Design and Applied Arts Index (DAAI) (ProQuest) | Architecture; Art; Design; Landscape Architecture; Urban Studies | - | Arts |
| EconLit (ProQuest) | Economics | - | Social Science |
| Film Index International (ProQuest) | Cinema and Film | - | Arts |
| Genetics Abstracts (ProQuest) | Genetics | - | Applied Science |
| GeoRef (Engineering Village) | Geology; Physics | - | Applied Science / Basic Science |
| Historical Abstracts (H.W. Wilson) | African Studies; Black Studies; Celtic Studies; French Studies; History; Islamic Studies; Italian Studies; Kinesiology; Middle Eastern Studies; Political Science; Renaissance; Slavic and East European Studies; Sports | - | Humanities |
| Humanities Abstracts (H.W. Wilson) | Humanities; History | - | Humanities |
| *Replacement for update:* Humanities Index (ProQuest) | Humanities; History | - | Humanities |
| Index Islamicus (ProQuest) | Archaeology; Islamic Studies; Middle Eastern Studies; South Asian Studies | - | Humanities |
| International Bibliography of Art (IBA) (ProQuest) | Architecture; Art; Medieval Studies | 1 (2%) | Arts |
| International Index to Music Periodicals (ProQuest) | Music | - | Arts |
| Linguistics and Language Behavior Abstracts (LLBA) (ProQuest) | Italian Studies; Linguistics | - | Humanities |
| MathSciNet | Engineering; Mathematics; Physics | - | Basic Science |
| MLA International Bibliography (ProQuest) | Celtic Studies; German Studies; Italian Studies; Linguistics; Literature; Medieval Studies; Mythology and Folklore; Portuguese Studies; Slavic and East European Studies; Spanish Studies | - | Humanities |
| Periodicals Archive Online (ProQuest) | Humanities; Social Sciences | - | Humanities / Social Science |
| Periodicals Index Online (ProQuest) | Humanities; Social Sciences | - | Humanities / Social Science |
| Physical Education Index (ProQuest) | Kinesiology; Physical Education; Recreation; Sports | - | Applied Science |
| ProQuest Religion (ProQuest) | Christianity; Religion | - | Humanities |
| Worldwide Political Science Abstracts (ProQuest) | Astronomy; International Relations; Slavic and East European Studies | - | Social Science |

Listed: 44 Literature Databases (40 original literature databases + 4 replacement literature databases)

^a^ Count may not total 45 and percentages may not total 100 due to records being found in the gray literature (9 records) and from scanning references (1 record)
^b^ Categories are not mutually exclusive, i.e., one article can appear in more than one literature database
^c^ Sources: 1) Economic and Social Research Council. Available at: http://www.esrc.ac.uk/about-us/what-is-social-science/social-science-disciplines. Accessed March 6, 2019. 2) Humanities Council of Washington, DC. What are the humanities? 2009. Available at: http://www.wdchumanities.org/docs/defininghumanities.pdf. Accessed March 6, 2019. 3) National Endowment for the Arts. Discipline descriptions. Available at: https://www.arts.gov/grants-organizations/discipline-descriptions. Accessed March 6, 2019. 4) AAMC. Basic Science. Available at: https://www.aamc.org/initiatives/research/334422/basicscience.html. Accessed March 6, 2019. 5) World Heritage Encyclopedia. Outline of applied science. Available at: http://www.worldheritage.org/articles/Outline_of_applied_science. Accessed March 6, 2019.
