# Supplementary material for: The views, perspectives, and experiences of academic researchers with data sharing and reuse: A meta-synthesis
Source: PLoS One. 2020 Feb 27;15(2):e0229182. doi: 10.1371/journal.pone.0229182 (PMC7046208; doi:10.1371/journal.pone.0229182)
Supplement: S5 Appendix — (DOCX) [file pone.0229182.s005.docx]

**S5 Appendix. Included studies.**

**INCLUDED STUDIES**

1. Allard S, Aydinoglu AU. Environmental researchers' data practices: An exploratory study in Turkey. International Symposium on Information Management in a Changing World. Springer Berlin Heidelberg. 2012; 317:13-24.
2. Bamkin M. Report of Findings from Focus Group and Online Questionnaire: The opinions of potential users of a policy databank service. JORD Project. 2014. Available at: https://jordproject.files.wordpress.com/2014/06/report-of-findings-from-focus-group-and-online-questionnaire.pdf. Accessed June 19, 2019.
3. Broom A, Cheshire L, Emmison M. Qualitative researchers' understandings of their practice and the implications for data archiving and sharing. Sociology. 2009; 43(6):1163-1180.
4. Carlson J, Stowell-Bracke M. Data management and sharing from the perspective of graduate students: an examination of the culture and practice at the water quality field station. portal: Libraries and the Academy. 2013; 13(4):343-361.
5. Cheah PY, Tangseefa D, Somsaman A, Chunsuttiwat T, Nosten F, Day NPJ, Bull S, Parker M. Perceived benefits, harms, and views about how to share data responsibly: a qualitative study of experiences with and attitudes toward data sharing among research staff and community representatives in Thailand. Journal of Empirical Research on Human Research Ethics. 2015; 10(3):278-289.
6. Colledge F, Persson K, Elger B, Shaw D. Sample and data sharing barriers in biobanking: consent, committees, and compromises. Annals of Diagnostic Pathology. 2014; 18(2):78-81.
7. Cragin MH, Palmer CL, Carlson JR, Witt M. Data sharing, small science and institutional repositories. Philosophical transactions. Series A, Mathematical, physical, and engineering sciences. 2010; 368(1926):4023-4038.
8. Delasalle J. Research data management at the University of Warwick: recent steps towards a joined-up approach at a UK university. LIBREAS. Library Ideas. 2013; 23. Available at: http://libreas.eu/ausgabe23/10delasalle. Accessed June 19, 2019.
9. Denny SG, Silaigwana B, Wassenaar D, Bull S, Parker M. Developing ethical practices for public health research data sharing in South Africa: the views and experiences from a diverse sample of research stakeholders. Journal of Empirical Research on Human Research Ethics. 2015; 10(3):290-301.
10. Diekemann AR, Wesolek A, Walters CD. The NSF/NIH effect: surveying the effect of data management requirements on faculty, sponsored programs, and institutional repositories The Journal of Academic Librarianship. 2014; 40:322-331.
11. Faniel IM, Jacobsen TE. Reusing scientific data: how earthquake engineering researchers assess the reusability of colleagues data. Computer Supported Cooperative Work. 2010; 19:355-375.
12. Faniel I, Kansa E, Kansa SW, Barrera-Gomez J, Yakel E. The challenges of digging data: A study of context in archaeological data reuse. Proceedings of the 13th ACM/IEEE-CS Joint Conference on Digital Libraries. 2013; 295-304.
13. Finn R, Wadhwa K, Taylor M, Sveinsdottir T, Noorman M, Sondervan J. Legal and ethical issues in open access and data dissemination and preservation. Recode Project. 2014. Available at: https://zenodo.org/record/1297492#.W3MvaM5KhhE. Accessed June 19, 2019.
14. Frank RD, Yakel E, Faniel IM. Destruction/reconstruction: preservation of archaeological and zoological research data. Archival Science. 2015; 15(2):141-167.
15. Hall N. Environmental studies faculty attitudes towards sharing of research data. Proceedings of the 13th ACM/IEEE-CS Joint Conference on Digital Libraries. 2013; 383-384.
16. Henty M, Weaver B, Bradbury SJ, Porter S. Investigating Data Management Practices in Australian Universities. 2008. Available at: http://eprints.qut.edu.au/14549. Accessed June 19, 2019.
17. Higman R, Pinfield S. Research data management and openness: the role of data sharing in developing institutional policies and practices. Program: Electronic Library and Information Systems. 2015; 49(4):364-381.
18. Hunt SL, Bakker CJ. A qualitative analysis of the information science needs of public health researchers in an academic setting. Journal of the Medical Library Association: JMLA. 2018 Apr;106(2):184.
19. Johnston L, Jeffryes J. Data management skills needed by structural engineering students: case study at the University of Minnesota. Journal of Professional Issues in Engineering Education and Practice. 2014; 140(2):05013002.
20. Johri A, Yang S, Vorvoreanu M, Madhavan K. Perceptions and practices of data sharing in engineering education. Advances in Engineering Education. 2016;5(2):n2.
21. Kervin K, Finholt T, Hedstrom M. Macro and micro pressures in data sharing. IEEE 13th International Conference. 2012; 525-32.
22. Kim Y, Stanton JM. Institutional and Individual Influences on Scientists’ Data Sharing Practices. Journal of Computational Science Education. 2012; 3(1):47-56.
23. Lage K, Losoff B, Maness J. Receptivity to library involvement in scientific data curation: a case study at the University of Colorado Boulder. portal: Libraries and the Academy. 2011; 11(4):915-937.
24. Manion FJ, Robbins RJ, Weems WA, Crowley RS. Security and privacy requirements for a multi-institutional cancer research data grid: an interview-based study. BMC Medical Informatics and Decision Making. 2009; 9:31.
25. Marcus C, Ball S, Delserone L, Hribar A, Loftus W. Understanding Research Behaviors, Information Resources, and Service Needs of Scientists and Graduate Students: A Study by the University of Minnesota Libraries. 2007. Available at: https://conservancy.umn.edu/handle/11299/5546. Accessed June 19, 2019.
26. McGuire AL, Achenbaum LS, Whitney SN, Slashinski MJ, Versalovic J, Keitel WA, McCurdy SA. Perspectives on human microbiome research ethics. Journal of Empirical Research on Human Research Ethics. 2012; 7(3):1-14.
27. McLure M, Level AV, Cranston CL, Oehlerts B, Culbertson M. Data curation: a study of researcher practices and needs. portal: Libraries and the Academy. 2014; 14(2):139-164.
28. Murillo AP. Data at risk initiative: examining and facilitating the scientific process in relation to endangered data. Data Science Journal. 2014; 12:207-219.
29. Noorman M, Kalaitzi V, Angelaki M, Tsoukala V, Linde P, Sveinsdottir T, Price L, Wessels B. Institutional barriers and good practice solutions. 2014. Available at: https://zenodo.org/record/1297494#.W3MwWc5KhhE. Accessed June 19, 2019.
30. Ochs M, Andrews C, Downs A, Morris-Knower J, Young S. Research practices and support needs of scholars in the field of agriculture at Cornell University. Journal of Agricultural & Food Information. 2017 Jul 3;18(3-4):200-19.
31. Oleksik G, Milic-Frayling N, Jones R. Beyond data sharing: Artifact ecology of a collaborative nanophotonics research centre. Proceedings of the ACM 2012 conference on Computer Supported Cooperative Work. 2012; 1165-1174.
32. Pepe A, Goodman A, Muench A, Crosas M, Erdmann C. How do astronomers share data? Reliability and persistence of datasets linked in AAS publications and a qualitative study of data practices among US astronomers. PloS One. 2014; 9(8):e104798.
33. Read KB, Surkis A, Larson C, McCrillis A, Graff A, Nicholson J, Xu J. Starting the data conversation: informing data services at an academic health sciences library. Journal of the Medical Library Association. 2015; 103(3):131-5.
34. Stamatolos A, Neville T, Henry D. Analyzing the Data Management Environment in a Master's-level Institution. Journal of Academic Librarianship. 2016; 42(2):154-160.
35. Stapleton S, Minson V, Spears L. Investigating the research practices of agriculture scholars: findings from the University of Florida. Journal of Agricultural & Food Information. 2017 Jul 3;18(3-4):327-46.
36. Sturges P, Bamkin M, Anders JHS, Hubbard B, Hussain A, Heeley M. Research Data Sharing: Developing a Stakeholder-Driven Model for Journal Policies. Journal of the Association for Information Science and Technology. 2014; 66(12):2445-2455.
37. Valentino M, Boock M. Data Management for Graduate Students: A Case Study at Oregon State University. Practical Academic Librarianship. 2015; 5(2):77-91.
38. Van den Eynden V, Bishop L. Sowing the seed: Incentives and motivations for sharing research data, a researcher’s perspective. 2014. Available at: http://www.data-archive.ac.uk/media/492924/ke_report-incentives-for-sharing-research-data.pdf. Accessed June 19, 2019.
39. Van Tuyl S, Michalek G. Assessing Research Data Management Practices of Faculty at Carnegie Mellon University. Journal of Librarianship and Scholarly Communication. 2015; 3(3):eP1258.
40. Wallis JC, Rolando E, Borgman CL. If we share data, will anyone use them? Data sharing and reuse in the long tail of science and technology. PLoS One. 2013; 8(7):e67332.
41. Williams SC. Data sharing interviews with crop sciences faculty: why they share data and how the library can help. Issues in Science and Technology Librarianship. 2013. Available at: http://www.istl.org/13-spring/refereed2.html. Accessed June 19, 2019.
42. Yatcilla JK, Bracke MS. Investigating the needs of agriculture scholars: the Purdue Case. Journal of Agricultural & Food Information. 2017 Jul 3;18(3-4):293-305.
43. Yoon A. End users’ trust in data repositories: definition and influences on trust development. Archival Science. 2014; 14(1):17-35.
44. Yoon A. Data reusers' trust development. Journal of the Association for Information Science and Technology. 2017 Apr;68(4):946-56.
45. Zimmerman AS. New knowledge from old data: the role of standards in the sharing and reuse of ecological data. 2008 Sep; 33(5):631-652.

**COMPANION ARTICLES**

1. Faniel IM. Data table. Email communication, 2016.
2. Williams SC. Using a bibliographic study to identify faculty candidates for data services. Science & Technology Libraries. 2013; 32(2):202-209.
3. Zimmerman AS. Data sharing and secondary use of scientific data: experiences of ecologists to locate data for reuse. PhD Dissertation. 2003.
