# Supplementary material for: The views, perspectives, and experiences of academic researchers with data sharing and reuse: A meta-synthesis
Source: PLoS One. 2020 Feb 27;15(2):e0229182. doi: 10.1371/journal.pone.0229182 (PMC7046208; doi:10.1371/journal.pone.0229182)
Supplement: S6 Appendix — (DOCX) [file pone.0229182.s006.docx]

## S6 Appendix. Included studies by discipline.

| **Discipline or area of study^a^** | **Included studies** | **Number of included studies identified,  *n* (%)^b^; *N* = 45** |
| --- | --- | --- |
|  |  |  |
| Combined  (multiple disciplines) | Allard 2012; Cragin 2010; Delasalle 2013; Finn 2014; Frank 2015; Henty 2008; Kervin 2012; Kim 2012; Lage 2011; Marcus2007; McLure 2014; Murillo 2014; Noorman 2014; Stamatolos 2016; van Tuyl 2015; Wallis 2013; Yoon 2016 | 17 (37%) |
| Agriculture and related subjects | Carlson 2013; Diekmann 2012; Williams 2013; Ochs 2017; Stapleton 2017; Yatcilla 2017 | 6 (13%) |
| Not reported | Bamkin 2014; Higman 2015; Sturges 2014; Valentino 2015; van den Eynden 2014 | 5 (11%) |
| Medicine & dentistry | Cheah 2015; Colledge 2014; Manion 2009; McGuire 2012 | 4 (8%) |
| Subjects allied to medicine | Denny 2015; Read 2015; Hunt 2018 | 3 (6%) |
| Engineering and technology | Faniel 2010; Johnston 2014; Johri 2016 | 3 (6%) |
| Physical sciences | Hall 2013; Oleksik 2012; Pepe 2014 | 3 (6%) |
| Social studies | Broom 2009; Yoon 2014 | 2 (4%) |
| Historical and philosophical studies | Faniel 2013 | 1 (2%) |
| Biological sciences | Zimmerman 2003 | 1 (2%) |

^a^ Classification system used is the HECoS (The Higher Education Classification of Subjects) (<https://www.hesa.ac.uk/innovation/hecos>). Discipline is reported directly by authors and abstracted from original studies.

^b^ Percentages may not total 100 because of rounding.
