# Supplementary material for: The views, perspectives, and experiences of academic researchers with data sharing and reuse: A meta-synthesis
Source: PLoS One. 2020 Feb 27;15(2):e0229182. doi: 10.1371/journal.pone.0229182 (PMC7046208; doi:10.1371/journal.pone.0229182)
Supplement: Attachment — Submitted filename: Response to Reviewers Comments.docx [file pone.0229182.s007.docx]

Response to Peer Reviewers

| **Reviewer #1 Comments** | **Author Response** |
| --- | --- |
| No statistical analysis was conducted in this study; however, the independent coders used the appropriate metric CASP to ensure that the documents were of high quality. However, surprisingly, the authors did not describe or include any intercoder reliability statistics for either the CASP coding nor the analytical themes coding. A discussion of this should be included in the study. | **Completed: Page 4-5**  We added a new section under Materials and Methods entitled ‘Types of Studies’ and provided the references and support to outline more information on metasynthesis as a study type. This includes the clarification that metasyntheses is a qualitative review and therefore does not use quantitative approaches/analysis, e.g., statistics (Walsh & Downe 2005). |
| Other comments:  Figure 2 was nearly impossible to see, a higher quality image needs to be provided. | **Completed: Figure 2**  The font size and dimensions have been increased on the image. |
| While this paper is well written and easy to follow, the findings leave the reading wanting to know why this study is important. It provides a summary of the other studies in data sharing, however, does not provide an understanding outside what is already known from the current data sharing research. I would suggest adding more to the discussion and conclusion to indicate why this study is important and what the academic community and data sharers, as well as repositories managers should learn from it. | **Completed: Page 3-4; Page 27; Page 29**  Page 3-4: A paragraph was added to the Introduction to clarify why this study is important.  Page 27, Last paragraph of Discussion: A paragraph was added to confirm why this study is important.  Page 29, Last sentence of Conclusions: The Conclusions were enhanced to clarify why this study is important. |

| **Reviewer #2 Comments** | **Author Response** |
| --- | --- |
| This is an interesting “meta-synthesis” of 45 studies previously published that addressed data sharing. The conclusions are not surprising, and the results (the ‘synthesis’) has all been published previously, as well as in review articles about the same topic. This is not novel, nor are new suggestions made for further work to be done or solutions to be tested. Nevertheless, it represents a comprehensive review of 45 studies. | **No Changes Made**  We searched for a review on this topic before undertaking our study but were not able to locate any. Reviewer #2 states “…the results (the ‘synthesis’) has all been published previously, as well as review articles about the same topic”.   - We would be very appreciative if the citations of the **qualitative** reviews on the perspectives/experiences of academic researchers and data sharing could be shared to determine if there is overlap with our study |
| While the authors have aggregated concerns and opinions about sharing data, they provide no statistical analysis of the frequency of those concerns. This is a major limitation of the work. How often did a concern or opinion need to be observed in the meta-analysis in order for it to be listed as a major concern? Did any concern(s) rise above others or for different types of research data? What weight should be assigned to the importance of each of these many concerns? | **Completed: Page 4-5**  We added a new section under Materials and Methods entitled ‘Types of Studies’ and provided the references and support to outline more information on metasynthesis as a study type. This includes the clarification that metasyntheses is a qualitative review and therefore does not use quantitative approaches/analysis, e.g., statistical analysis, meta-analysis, or weighting, i.e., quantitative approaches (Zimmer 2006; Walsh & Downe 2005; Sandelowski & Barroso 2007). |
| While a majority of the studies utilized interviews as the principal methodology, 8 studies did not. Were there any differences between focus groups (or other) and interview results? Were any surveys found? | **Completed: Page 4-5**  We added a new section under Materials and Methods entitled ‘Types of Studies’ and provided the references and support to outline more information on metasynthesis as a study type. This includes the clarification that metasyntheses is a qualitative review and therefore does not use quantitative approaches/analysis, e.g., subgroup analyses (Zimmer 2006; Walsh & Downe 2005; Sandelowski & Barroso 2007).  Surveys are eligible if they included mixed methods and is described on Page 5-6 under Eligibility Criteria: “Mixed methods studies that used both qualitative and quantitative methods within the same study were eligible if the qualitative portion met our inclusion criteria.” |

| The authors appear to comingle studies that interviewed (or reported on focus groups of) researchers that are required to (or encouraged to) deposit data as well as attitudes of others (e.g. data scientists, students, data users, lawyers, etc.). One would anticipate that the opinions of each of these would be quite different, and different again from participants in clinical trials or research. How these different subgroups weighted their concerns, and how analyzed should be discussed. This may require incremental analysis by the authors. | **Completed: Page 4-5**  We added a new section under Materials and Methods entitled ‘Types of Studies’ and provided the references and support to outline more information on metasynthesis as a study type. This includes the clarification that metasyntheses is a qualitative review and therefore does not use quantitative approaches/analysis, e.g., subgroup analyses (Zimmer 2006; Walsh & Downe 2005; Sandelowski & Barroso 2007).  We stated on Page 5 that “50% or more of the total sample had to be researchers from academic institutions in order to be eligible for inclusion”. As a result, the majority of participants being analysed would be researchers (i.e., the other participants would be present in lesser numbers). |
| --- | --- |
| It is surprising to this reviewer that there is no differentiation between the attitudes of scientists in the different fields: Investigators in some research disciplines are accustomed to sharing data (e.g. astronomy, genetics) and have standards in place for doing so. Researchers in other disciplines, most notably in human participant research where issues of privacy and confidentiality are central concerns, appear to drive reticence to share. The authors state that 17 of 45 studies were from “mixed” disciplines; nevertheless, this reviewer requests an analysis by type of research, and by ‘concern’. | **Completed: Page 4-5**  We added a new section under Materials and Methods entitled ‘Types of Studies’ and provided the references and support to outline more information on metasynthesis as a study type. This includes the clarification that metasyntheses is a qualitative review and therefore does not use quantitative approaches/analysis, e.g., sub-group analysis such as by type of research and by ‘concern’ (Walsh & Downe 2005). |
| The authors mention that one of the authors “conducted focus groups with academic researchers…and found that data saturation was reached after conducting four focus groups despite collecting data from discrete disciplines (i.e., health science, humanities, natural science).” How did that research factor into this current analysis and what significance does it hold? | **Completed: Page 27-28**  Further explanation has been provided to this statement in order to provide clarification that despite different research methods and processes being used in various disciplines, researchers aligned with issues in research data management. |
| Did the authors perceive any differences in opinions and attitudes over time? | **Completed: Page 4-5**  We added a new section under Materials and Methods entitled ‘Types of Studies’ and provided the references and support to outline more information on metasynthesis as a study type. This includes the clarification that metasyntheses is a qualitative review and therefore does not use quantitative approaches/analysis (Glenton 2014). |
| Figure 2 is illegible. Why not present in a tabular form? | **Completed: Figure 2**  The font size and dimensions have been increased on the image. |

Glenton C, Lewin S. Using evidence from qualitative research to develop WHO guidelines. WHO Handbook for Guideline Development. 2nd Edition. Geneva: WHO, 2014.

Sherwood, G. (1999). Meta-synthesis: Merging qualitative studies to develop nursing knowledge. International

Journal for Human Caring, 3, 37-42

Sandelowski M, Barroso J. Handbook for synthesizing qualitative research. New York, NY: Springer, 2007.

Walsh D, Downe S. Meta-synthesis method for qualitative research: A literature review. Journal of Advanced Nursing. 2005; 50:204-211.

Zimmer L. Qualitative meta-synthesis: A question of dialoguing with texts. Journal of Advanced Nursing. 2006; 53:311-318.
